# Discovery and characterization of olokizumab: A humanized antibody targeting interleukin-6 and neutralizing gp130-signaling

**DOI:** 10.4161/mabs.28612

**Published:** 2014-04-02

**Authors:** Stevan Shaw, Tim Bourne, Chris Meier, Bruce Carrington, Rich Gelinas, Alistair Henry, Andrew Popplewell, Ralph Adams, Terry Baker, Steve Rapecki, Diane Marshall, Adrian Moore, Helen Neale, Alastair Lawson

**Affiliations:** 1 UCB Pharma; Berkshire, UK; 2 Institute for Systems Biology; Seattle, WA USA

**Keywords:** interleukin 6, IL-6, antibody, site 3, gp130, olokizumab, neutralization, structure

## Abstract

Interleukin-6 (IL-6) is a critical regulator of the immune system and has been widely implicated in autoimmune disease. Here, we describe the discovery and characterization of olokizumab, a humanized antibody to IL-6. Data from structural biology, cell biology and primate pharmacology demonstrate the therapeutic potential of targeting IL-6 at “Site 3”, blocking the interaction with the signaling co-receptor gp130.

## Introduction

Interleukin-6 (IL-6) is a pleiotropic cytokine that plays a central role in immune regulation and inflammation.^1^^,^^2^ Its importance in moderating both innate and adaptive immune responses is evidenced by the broad array of cells that secrete this cytokine, including monocytes, macrophages, T cells, and B cells.^3^^,^^4^ IL-6 directs chemokine-regulated trafficking of leukocytes and induces proliferation and differentiation of T cells, as well as antibody production by B cells. IL-6 also contributes to the transition from innate to adaptive immunity through the regulation of leukocyte activation, differentiation, and proliferation.^4^^,^^5^

IL-6 interacts with two receptors, gp80 (also known as IL-6 receptor [IL-6R], CD126) and the signal-transducing co-receptor molecule gp130 (CD130),^1^ to form a hexameric signaling complex. Formation of this signaling complex is thought to be a stepwise process during which the IL-6 molecule first binds to gp80 at Site 1 to form a dimer, and subsequently to gp130 at Site 2 to form a heterotrimer. Two heterotrimers then combine to form the final active hexameric signaling complex (gp80:IL-6:gp130)_2_ through interaction between Site 3 on IL-6 and domain 1 of gp130.^6^^,^^7^ The order of IL6:gp80 complex interactions with gp130 is controversial, as the higher affinity of the Site 3 vs. Site 2 interaction suggests that heterotrimer formation may actually be driven via Site 3.^8^ The full hexameric complex is required for effective IL-6 signaling, and neither the IL-6:gp80 dimer nor the gp80:IL-6:gp130 trimer is able to initiate signal transduction.

IL-6 signaling can be mediated via a signaling complex that incorporates either membrane-bound gp80 (*cis*-signaling) or a soluble form of gp80 (*trans*-signaling), which is produced as a result of proteolytic cleavage or alternative splicing.^1^^,^^2^^,^^9^ While gp130 is ubiquitously expressed, gp80 is present only on certain leukocyte subsets and hepatocytes.^10^ Soluble gp80-mediated *trans*-signaling therefore enables IL-6-driven stimulation of cells that do not express gp80, thereby expanding the range of cell types capable of responding to IL-6. In addition, some activated immune cells, such as T cells, progressively lose their ability to respond to IL-6 *cis*-signaling because they shed their membrane-bound gp80; *trans*-signaling is thought to maintain these effector T cells in an activated state over prolonged periods of time.^4^

Both *cis*- and *trans*-signaling induce an intracellular signaling cascade following tyrosine phosphorylation on the cytoplasmic tail of gp130,^1^^,^^2^ with subsequent activation of downstream factors, including the Janus-activated kinases (JAK) and the signal transduction and activators of transcription (STATs).^11^ IL-6 also regulates the expression of acute phase proteins via Ras-Raf and subsequent phosphorylation of mitogen-activated protein kinase (MAPK).^2^

Given its critical immunoregulatory function, it is not surprising that IL-6 signaling is implicated in the progression of several autoimmune diseases, including rheumatoid arthritis (RA),^12^ Crohn disease,^13^ and systemic lupus erythematosus.^14^^,^^15^ During acute inflammation in RA, IL-6 is released by monocytes, macrophages, and endothelial cells.^10^ Stimulation of B cells by IL-6 leads to increased levels of polyclonal γ-globulins and rheumatoid factor. T-cell activation by IL-6 in the presence of autoantigens induces differentiation along the T_h_2 pathway^2^ and the activation of autoreactive T_h_17 cells.^2^^,^^12^ In the synovium, infiltration of inflammatory cells contributes to pannus formation (an abnormal layer of fibrovascular tissue), ultimately leading to erosion of cartilage and adjacent bone.^12^ IL-6 also promotes the production of vascular endothelial cell growth factor (VEGF), which leads to angiogenesis and in turn drives further bone destruction.^16^

In patients with Crohn disease, high concentrations of IL-6 are present in the serum and intestinal tissue.^13^ IL-6, in conjunction with other cytokines, upregulates endothelial expression of vascular cell-adhesion molecule 1 (VCAM1), very late antigen 4 (VLA4), and intercellular adhesion molecule 1 (ICAM1) in the small intestine. This leads to recruitment of circulating effector inflammatory cells into the endothelium and potentiation of the inflammatory cycle.^17^ Concentrations of both IL-6 and soluble gp80 are strongly correlated with C-reactive protein, a marker of inflammation, and levels of this protein are higher in serum from patients with active disease than in those with inactive disease.^18^

In systemic lupus erythematosus, the exact mechanism by which IL-6 contributes to the disease pathology is not yet known; however, elevated serum IL-6 has been shown to correlate with B-cell hyperactivity and autoantibody production.^19^ Addition of anti-IL-6 monoclonal antibodies has been shown to reduce these effects.^19^^,^^20^

An increasing body of evidence suggests that *trans*-signaling is the crucial driver of IL-6-mediated pathology in autoimmune conditions.^9^ For example, elevated concentrations of both IL-6 and soluble gp80 in the synovial fluid from patients with RA are correlated with the severity of inflammation and joint destruction.^10^ The generation and activation of osteoclasts (critical mediators of bone erosion that are present in excess in RA synovium) have been shown to be stimulated by IL-6 in patients with RA.^16^ Osteoclast formation from fibroblast-like synovial cells requires the presence of both IL-6 and soluble gp80, indicating a crucial role for *trans*-signaling in disease progression.^16^^,^^21^ Furthermore, both endothelial cells and synoviocytes are implicated in synovial angiogenesis in RA; however, these cells do not express gp80.^5^^,^^10^^,^^16^^,^^22^^,^^23^ In addition, the observation that specific inhibition of soluble gp80 leads to apoptosis in T cells from patients with Crohn disease, and improves intestinal inflammation in an experimental colitis model,^24^ is indicative of a role for *trans*-signaling in maintaining the adverse effects associated with these conditions. The critical role of IL-6 *trans*-signaling in mediating autoimmune pathology suggests that the modulation of IL-6 biology on the gp130 axis is a valid therapeutic approach to treat a range of diseases.

Biologic-based therapeutic strategies include targeting the IL-6 receptor, gp80 (tocilizumab,^25^ sarilumab^26^ and ALX-0061^27^), targeting IL-6 itself (olokizumab,^28^ sirukumab,^29^ siltuximab,^30^ clazakizumab,^31^ PF-04236921^32^ and the AMG-220 avimer^33^), and targeting both components with a gp130-Fc fusion protein (FE999301).^34^

Here, we present the discovery, characterization and in vivo performance of olokizumab, a therapeutic antibody that binds to IL-6 at Site 3 and neutralizes biological activity through blocking hexamer formation on the gp130 signaling axis.

## Results

### Discovery, humanization and characterization of olokizumab

Monoclonal anti-human IL-6 antibodies were generated from immunized rats by cloning variable region genes from isolated B cells (Fig. 1). Following rigorous screening of some 7x10^8^ B cells, antibody 132E09 was selected for humanization from a panel of 20 antibodies, based on sequence, affinity, and neutralization of the biological activity of IL-6. In order to retain full activity, residue 49 of the rat heavy chain was retained in the humanized antibody. Alignments of the rat antibody (donor) sequence with the human germline (acceptor) frameworks are shown in Figure 2, together with the humanized sequence of olokizumab.

**Figure:**
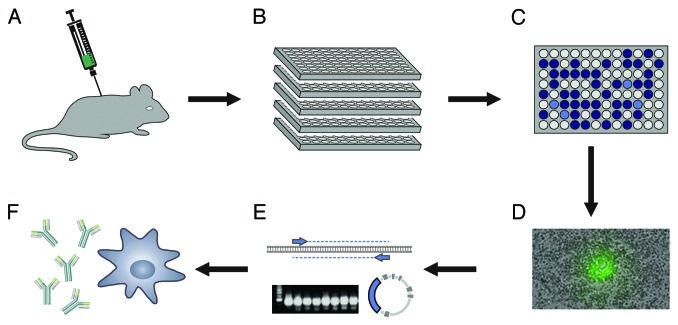
**Figure 1. Generation of anti-IL-6 antibodies.** (**A**) Rats were immunized by subcutaneous injection of recombinant human IL-6. (**B**) Immune rat lymphocytes were cultured in 96-well plates. (**C**) Supernatants were screened for neutralizing anti-IL-6 antibodies in ELISA and cell assays. (**D**) Individual B cells secreting specific antibody were picked. (**E**) Heavy and light chain variable regions were cloned from single cells and transferred to an IgG format. (**F**) Recombinant IgG antibodies were expressed.

**Figure:**
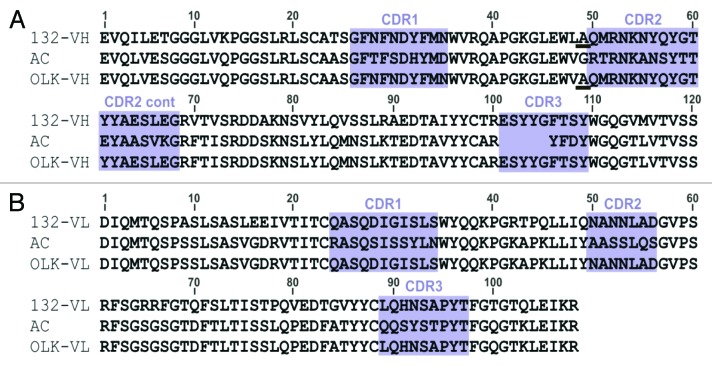
**Figure 2. Humanization of antibody 132E09 to generate olokizumab.** (**A**) Humanization of the heavy chain variable region. The human VH3 1–4 3–72 V-region with the JH4 J-region (V BASE, http://vbase.mrc-cpe.cam.ac.uk/) was chosen as the heavy-chain germline acceptor sequence. 132-VH = heavy-chain sequence of antibody 132E09; AC = human IgG γ4 acceptor sequence; OLK-VH = olokizumab heavy-chain sequence. (**B**) Humanization of the light-chain variable region. The human VK1 2–1-(1) O12 V-region with the JK2 J-region (V BASE, http://vbase.mrc-cpe.cam.ac.uk/) was chosen as the light-chain germline acceptor sequence. 132-VL = light-chain sequence antibody 132E09; AC = human κ light-chain acceptor sequence; OLK-VL = olokizumab light-chain sequence.

Olokizumab bound human IL-6 with an affinity (KD) of 10 pM, (ka = 7.9 × 10^5^ M^−1^ s^−1^ [n = 2]; kd = 7.7 × 10^−6^ s^−1^ [n = 4]) in a Biacore with captured antibody and solution phase IL-6. There was no discernible drop-off in the dissociation rate against cynomolgus IL-6.

### Crystal structure of olokizumab/IL-6 complex

To determine the epitope and understand the mode of action of olokizumab further, the crystal structure of the Fab portion of the antibody in complex with IL-6 was determined. The structure (resolution 2.2Å; Fig. 3) shows a 1:1 complex between IL-6 and the Fab fragment of the antibody.

**Figure:**
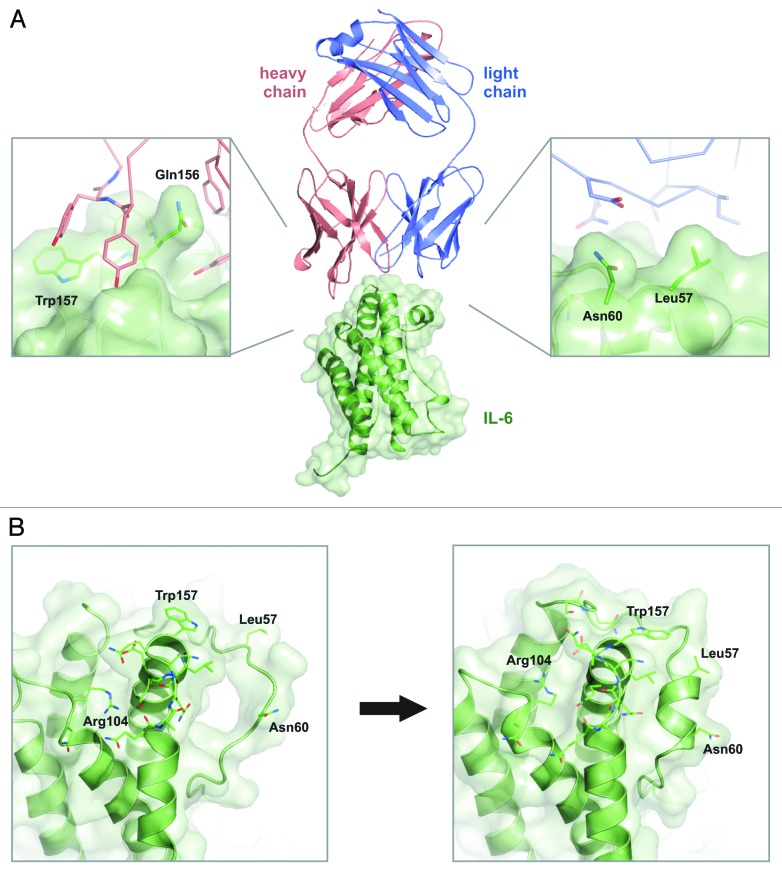
**Figure 3. Crystal structure of the Fab portion of olokizumab in complex with IL-6.** (**A**) The crystal structure of the antibody:target complex. The heavy chain is shown in pink, the light chain is shown in blue, and IL-6 is shown in green. Critical interactions between the heavy chain and residues of IL-6 are highlighted in the left panel; important interactions between the light chain and IL-6 and shown in the right panel. (**B**) Conformational changes in IL-6 on binding olokizumab. The left panel shows IL-6 when bound to its receptors,^6^ the right panel shows IL-6 when bound to olokizumab. For clarity, IL-6 is shown in isolation (i.e., without bound receptors or antibodies). There are substantial conformational changes, especially residues 50–60 (which go from a random coil conformational to an α-helix conformation), residue trp157 (in which the indole ring flips ~180°), and residue arg104 (which adopts a different rotameric conformation). As a result of these conformational changes, the gp130 binding pocket, which is located between these residues, becomes occluded when bound to olokizumab.

The epitope of olokizumab lies in the gp130-binding region of IL-6. The IL-6 residue trp157, which is known to be a critical mediator of the IL-6:gp130 domain 1 interaction,^6^ makes extensive hydrophobic contacts with tyrosine and phenylalanine side chains from the heavy chain of the antibody (Fig. 3A).

The interface between olokizumab and IL-6 is highly interdigitated; most importantly, residue gln156 protrudes from the surface of IL-6 into a pocket formed by the CDRs of the antibody. There are numerous direct contacts between IL-6 and olokizumab, including 19 hydrogen bonds,^35^ as well as several contacts mediated by buried water molecules. These suggest that the interaction between olokizumab and IL-6 is both entropically and enthalpically driven.^36^

In the complex, the overall structures of both olokizumab and IL-6 are as expected, with IL-6 forming the four-helix bundle structure reported previously^7^ and olokizumab showing the typical immunoglobulin structure of antibodies. However, when we compared the structure of IL-6 bound to olokizumab with that of IL-6 in its signaling state,^6^ we found several unusual conformational differences (Fig. 3B), i.e., IL-6 residues arg104 and trp157 underwent significant side chain rearrangements. Even more strikingly, IL-6 residues 50–60 went from a random coil conformation (in the receptor-bound signaling state, PDB 1P9M) to a helical conformation (in the antibody-bound state), with the light chain of olokizumab interacting with leu57 and asn60. As a result of these conformational differences, the gp130 receptor-binding pocket (located on IL-6 between trp157 and residues 50–60) is occluded and structurally closed when IL-6 is bound to olokizumab.

The crystal structure of the Fab portion of olokizumab in complex with IL-6 strongly suggests that the mode of action of olokizumab involves steric blockade of the interaction between IL-6 and domain 1 of gp-130.

### Neutralization activity of olokizumab

Olokizumab exhibited potent neutralization of both *cis*- and *trans*- signaling of IL-6 (Fig. 4). Neutralization of *cis* signaling (Fig. 4A), with C-reactive protein (CRP) and serum amyloid A (SAA) readouts, was demonstrated in primary human hepatocytes, which express membrane gp80, while neutralization of *trans* signaling (Fig. 4B) was shown in a human umbilical vein endothelial cell (HUVEC) system, in which soluble gp80 was required to be added for phosphorylation of STAT3 to occur. Neutralization was seen at close to stoichiometric equivalence.

**Figure:**
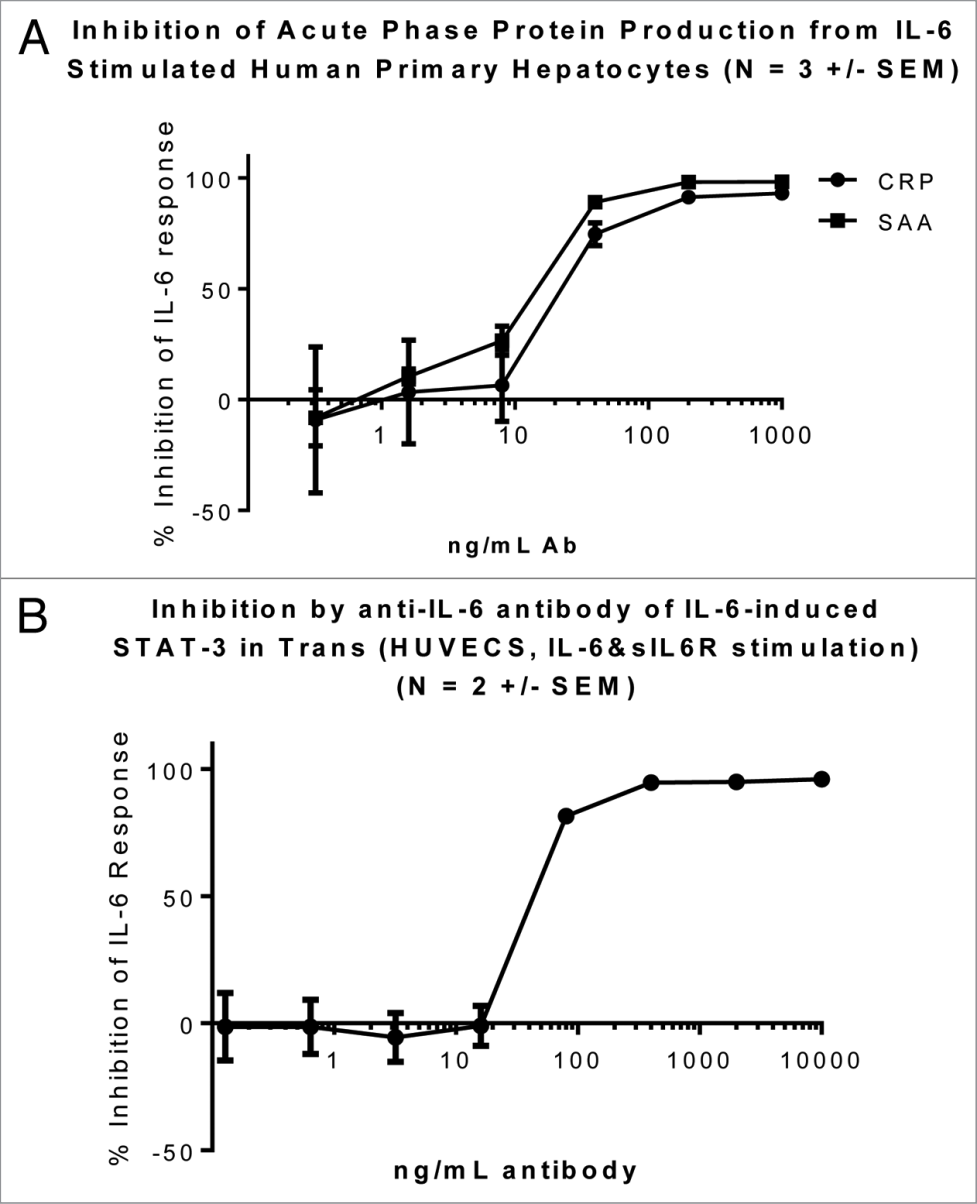
**Figure 4. Olokizumab in cell assays to assess neutralization of IL-6 activity.** (**A**) Cis signaling Primary human hepatocytes were cultured in collagen-coated plates and stimulated with IL-6 (12.5 ng/mL) in the presence or absence of olokizumab (titration from 10 µg/mL) for 72 h. Cell supernatants were analyzed for acute phase proteins CRP and SAA using a Luminex kit. Data are plotted with standard errors of means from triplicate determinations in three experiments. (**B**) Trans signaling Different concentrations of olokizumab were pre-incubated with IL-6 at 25ng/mL followed by addition of soluble IL-6 receptor gp80 at 125 ng/mL. This complex was added to prepared HUVEC cells and incubated at 37 °C for 20 min to allow IL-6-induced STAT-3 phosphorylation to occur. The activation was stopped by the addition of ice-cold lysis buffer, and cell supernatants were analyzed for STAT3 phosphorylation using a MSD STAT-3 kit. Data are plotted with standard errors of means from triplicate determinations in two experiments.

### Olokizumab in primate arthritis model

To assess the in vivo efficacy of olokizumab, the antibody was tested in a cynomolgus collagen-induced arthritis model, which measures various signs and symptoms associated with disease severity (Fig. 5). Compared with the control, substantially reduced arthritis scores (Fig. 5A), and CRP levels (Fig. 5B), with improvements in histology (Fig. 5C) and bone erosion scores (Fig. 5D), were observed with a dose of 20 mg/kg of olokizumab. These results indicated that olokizumab could potently suppress signs and symptoms of arthritis in vivo, and at the 20 mg/kg dose the reduction in arthritis score was statistically significant (Wilcoxon rank sum test).

**Figure:**
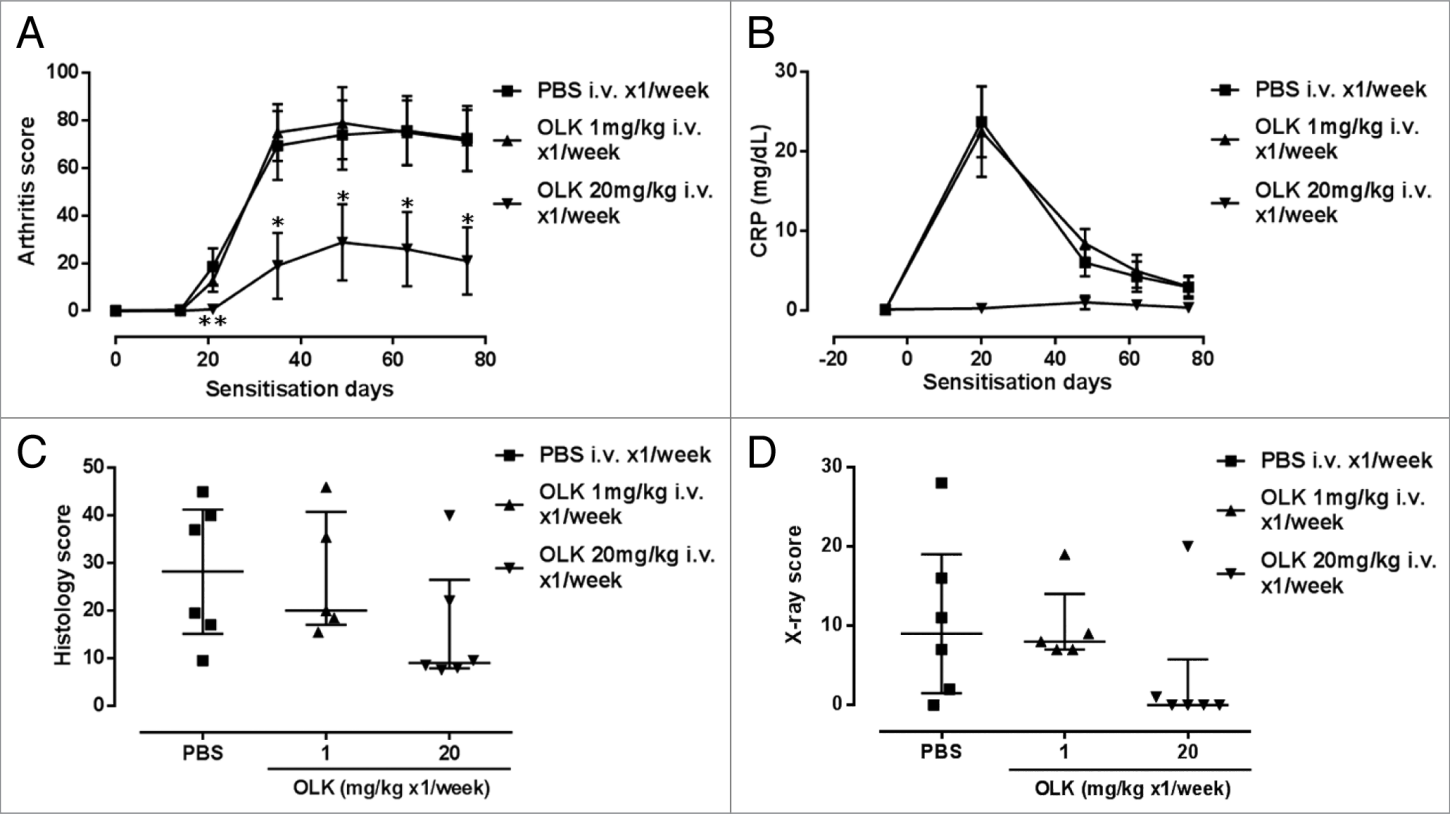
**Figure 5.** Olokizumab in a cynomolgus monkey collagen-induced arthritis model. Arthritis was induced in female monkeys by two sensitizations with bovine type II collagen in Freund’s complete adjuvant separated by a period of 3 wk. There were six monkeys in the PBS group, five in the OLK 1 mg/kg group and six in the OLK 10 mg/kg group. (**A**) Arthritis score. Arthritis score was assessed in a blinded manner by examination of the swelling of the metacarpophalangeal, proximal interphalangeal, distal interphalangeal joints, the wrist, ankle, elbow, and knee joints. Each joint was given a score from 0 (no abnormality) to 4 (rigidity of the joints). Arthritis score for each animal was the total of the joint scores. The following scale was used to score the arthritis disease progression in joints: 0 = no abnormality; 1 = swelling not visible, but can be determined by touch; 2 = swelling just visible and can be confirmed by touch; 3 = swelling clearly visible; and 4 = rigidity of the joints. The maximum score per animal was 256. The reduction in arthritis score in the 20mg/kg group was statistically significant (*P* < 0.01 [**], and *P* < 0.05 [*]; Wilcoxon rank sum test). (**B**) Effect of olokizumab on C-reactive protein. CRP was measured by latex-enhanced turbidimetric immunoassay in an automatic analyzer. (**C**) Histology score. Hyperplasia, granulation tissue, fibrosis, and cartilage and bone destruction were assessed after treatment with olokizumab to determine a composite histology score. Right carpal joint and PIP joints of right limb (total 5 joints) were examined. Paraffin embedded tissue, was cut and stained with hematoxylin-eosin and safranin-O. Each joint was scored for synovial hyperplasia (0–2), granulation tissue (0–2), fibrosis (0–2), degeneration of joint cartilage (0–0.5), osteoclasia (0–2) and osteogenesis (0–1). Histology score for each animal was the total of all the joint scores. The errors are interquartile ranges about the median. (**D**) X-ray score of bone erosion. Effect of olokizumab on joint degradation in collagen-induced arthritis model. A total of 48 joints were examined for signs of erosion after treatment with olokizumab. Joints were scored accordingly; sign of joint erosion = 1 and no erosion = 0.The total number of joints in the proximal, middle and distal digital phalanges of the forelimb and hindlimb (second, third, fourth, and fifth digits) were assessed. When bone erosion was noted, 1 point was added to the score. The X-ray score for each animal was the total of the joint scores. The errors are interquartile ranges about the median.

## Discussion

Data from molecular structural studies, cell assays and in vivo models indicate that targeting the IL-6:gp130 axis by blockade of Site3 on IL-6 represents a favorable point of pharmaceutical intervention in autoimmune disease, and strongly support the ongoing clinical evaluation of olokizumab.

The 132E09 antibody variable region was selected following an ultra-high throughput screening campaign, in which antibodies from up to one billion B cells from immunized animals were assessed for potency and ability to modulate the biological activity of IL-6. By screening in this manner, with no pre-conceived notions about an optimal point of intervention, and by incorporating information from structural studies of antibody/IL-6 complexes at an early stage, we found that the data were guiding us to Site 3.

Historically, drug discovery has focused on the identification and validation of molecular targets.^37^ However, our work illustrates that, even for well-validated targets such as IL-6, there is scope for further refinement in the choice of therapeutic axis, which may be critical in the context of disease outcome. As biopharmaceuticals are used to target ever more complex biologic processes (e.g., disrupting multi-receptor systems; simultaneously modulating multiple signaling pathways; tackling novel classes of targets), identifying not just the target but the optimal axis on the target can be expected to become increasingly important.^38^ Combining a molecular and structural perspective with in vivo pharmacology is a particularly powerful approach in this endeavor.

Our work shows that the ability of antibodies to bind to proteins (and the consequences of that binding) can be dependent on the conformational structure of both binding partners. A recent study has shown that antibodies can undergo substantial conformational changes when binding to target proteins.^39^ It has also been suggested that antibodies induce conformational changes, causing functional modification of target proteins (both inhibition and enhancement).^40^ However, only a few studies^41^^,^^42^ have so far provided direct evidence of such conformational changes. Our study has shown that the conformational structure of IL-6 in complex with olokizumab is substantially different to that of IL-6 bound to gp130,^6^ which may provide additional rationale for the potent anti-inflammatory effects of olokizumab.

## Materials and Methods

### Discovery and humanization of olokizumab

Anti-IL-6-binding antibodies were isolated using UCB's proprietary antibody discovery platform,^43^^,^^44^ which is outlined in Figure 1. Rats were immunized by subcutaneous injection of recombinant human IL-6 (Peprotech, catalog number 200–06). Spleens were harvested 1–2 wk after the last immunization, and single-cell suspensions were prepared. Immune rat lymphocytes were cultured in the presence of irradiated mouse thymoma EL4 cells and rabbit T-cell-conditioned media for 1 wk in 96-well microtiter plates (in total, 1,500 such plates were set up, with some 5000 B cells/well). Supernatants were screened for the presence of antibodies specific for human IL-6 in an enzyme-linked immunosorbent assay (ELISA), in which ~2500 wells showed antibody binding. These antibodies were further screened for the ability to neutralize both cis and trans biological effects of human IL-6 in a T1165-based bioassay, leading to the identification of ~250 positive candidates.^45^

Approximately 250 individual, specific B-cells were isolated from microtiter wells, and heavy and light chain variable region genes were cloned from each. Variable regions were expressed in recombinant IgG format, and recombinant antibodies were tested for binding affinity to IL-6 in surface plasmon resonance experiments (General Electric Biacore instrument).

Antibody 132E09 was selected for humanization by grafting the sequence of the complementarity-determining regions (CDRs) onto human IgG-γ4 germline frameworks.

The CDRs were grafted from the donor to the acceptor sequence according to the definition of Kabat et al.,^46^ with the exception of CDR-H1 for which the combined Chothia/Kabat definition was used.^47^ In order to retain full activity, donor residue 49 was retained in the humanized heavy chain. The resulting humanized antibody was referred to as CDP6038 during development and is now known as olokizumab.

### Crystallization and structure determination of the Fab portion of olokizumab in complex with human IL-6

To understand the binding and likely mode of action of olokizumab, we determined the crystal structure of the Fab portion of the antibody in complex with IL-6. Human IL-6 was expressed in *E. coli* Origami B cells (catalog number 71408, Novagen) and purified using a combination of affinity and size-exclusion chromatography. The Fab portion of olokizumab was expressed transiently in mammalian cells via established protocols,^48^ and was purified using KappaSelect resin (GE Healthcare, catalog number 17–5458–11). Purified IL-6 and the Fab portion of olokizumab were mixed at a stoichiometry of 1:1, and the complex was purified by size-exclusion chromatography before concentration. Diffraction quality crystals grew within 8 d in 800 nL sitting drops (containing 4 mg/mL complex, 0.1 M ammonium sulfate, 50 mM MES buffer pH 6.5, 11% polyethylene glycol 8000) equilibrated against a reservoir solution of 0.2 M ammonium sulfate, 100 mM MES pH 6.5, and 22% polyethylene glycol 8000. Crystals were harvested, transferred to cryoprotection buffer consisting of 80% reservoir solution mixed with 20% glycerol, and flash frozen in liquid nitrogen (–180 °C). Diffraction data to 2.2 Å resolution were collected at Diamond Light Source, and processed using the computer programs MosFlm and Scala.^49^ Crystallographic data collection and processing statistics are shown in Table 1. The structure was solved by molecular replacement using the program Phaser.^50^ Rebuilding and refinement was performed using the CCP4 suite of programs (Collaborative Computational Project Number 4 1994), Refmac^51^ and Coot.^52^ Molecular visualizations were generated with PyMOL.^53^ The final model had an *R*-factor of 17.6% and a free *R*-factor of value of 21.5%. Table 1 shows full refinement and model-building statistics.

**Table:** **Table 1.** Data collection and refinement statistics

**Data collection and processing statistics**	
Experiment type	Single-wavelength
X-ray source	Diamond light source, beamline I02
Wavelength (Å)	0.9795
Resolution limits (Å)	35.00–2.20 (2.32–2.20)
Space group	P6
Unit cell parameters (Å,°)	a = b = 241.99 c = 76.59
	α = β = 90 γ = 120
Number of unique reflections	126842 (18445)
Completeness (%)	98.1 (98.0)
*I/σ* (I)	10.8 (4.2)
*R*_merge_ (%)*	11.9 (33.8)
**Refinement statistics**	
Resolution limits (Å)	30.00–2.20 (2.26–2.20)
*R*-factor (%)	17.6
Free *R*-factor (%)	21.5
Number of non-H atoms^†^	9396 (protein), 1578 (water), 25 (ions)
R.m.s.d. bond length (Å)	0.008
R.m.s.d. bond angles (°)	1.115
Mean B value (Å^2^)	23
**Ramachandran plot^‡^**	
Residues in allowed region (%)	99.7
Residues in disallowed region (%)	0.3

Values in parentheses refer to the highest resolution shell. The crystals of the olokizumab-Fab:IL-6 complex diffracted well, as indicated. However, they proved to be sensitive to X-ray radiation damage – this is not uncommon in crystals of antibody Fab fragments. To take account of the radiation damage, the atoms most affected (mainly sulfur atoms, which form part of cysteine residues) were modeled to reduced occupancy, as suggested in the literature.^54^ *R_merge_ = ∑|*I_j_* – < *I* > | / *I_j_*, where *I_j_* is the intensity of an individual observation of a reflection and < *I* > is the average intensity of that reflection. ^†^The crystallographic asymmetric unit contains two copies of the olokizumab-Fab:IL-6 complex. ^‡^Calculated using the program MolProbity.^55^ R.m.s.d. = root mean squared deviations.

### Database accession codes

Coordinates and structure factors of the Fab portion of olokizumab in complex with human IL-6 have been deposited in the Protein Data Bank (PDB) with accession code 4CNI.

### Neutralization activity of olokizumab

#### Cis signaling

Primary human hepatocytes (Life Technologies, catalog number HMCSPL) were allowed to adhere to collagen-coated plates (Invitrogen, catalog number A1142803) in Williams media (Invitrogen, catalog number 12551032) with Glutamax (Invitrogen, catalog number 32551020), 10% fetal calf serum and antibiotics for 24 h, before addition of IL-6 (Peprotech) or olokizumab, followed by further culture for 72 h. Cell supernatants were analyzed for acute phase proteins CRP and SAA using a Luminex kit (Millipore, catalog number HCVD2–67BK-03).

#### Trans signaling

Anti-IL-6 antibody at 12.5ng/ml was pre-incubated with IL-6 (R&D Systems, catalog number 206-IL-050) @ 25ng/mL for 20 min at room temperature, followed by addition of soluble IL-6 receptor gp80 (R&D Systems, catalog number 227-SR-025) at 125 ng/mL. This complex was then added to prepared HUVEC cells and incubated at 37 °C for 20 min to allow IL-6-induced STAT-3 phosphorylation to occur. The activation was stopped by the addition of ice-cold lysis buffer, and cell supernatants were analyzed for STAT3 phosphorylation with Sulfo-tagged™ anti-STAT-3 antibody (Meso Scale, catalog number K150SVD) using a MSD SECTOR™ Imager 6000 reader.

For both assays, an absolute IC_50_ calculation was used and the X data was log transformed before IC_50_s were calculated. Fifty = 50, Top = 100, Y = Bottom + (Top-Bottom)/(1+10^[{LogIC_50_ – X}*HillSlope + log{Top-Bottom}/{Fifty-Bottom} − 1]).

All calculations were performed using Graphpad Prism version 6.1.

### Collagen-induced arthritis model in cynomolgus monkeys

This study was performed at SNBL in Japan. Arthritis was induced in female monkeys by two sensitizations with bovine type II collagen in Freund’s Complete Adjuvant, separated by a period of 3 wk. Olokizumab was administered intravenously once a week at 1 or 20mg/kg.

### Arthritis score

Arthritis score was assessed in a blinded manner by examination of the swelling of the metacarpophalangeal, proximal interphalangeal, distal interphalangeal joints, the wrist, ankle, elbow, and knee joints. Each joint was given a score from 0 (no abnormality) to 4 (rigidity of the joints). Arthritis score for each animal was the total of the joint scores. Wilcoxon rank sum statistical analysis was performed.

### C-reactive protein

CRP was measured by latex-enhanced turbidimetric immunoassay in an automatic analyzer (JCA-BM8, JEOL Co., Ltd.).

### Histology score

Right carpal joint and PIP joints of right limb (total 5 joints) were examined. Paraffin embedded tissue, was cut and stained with hematoxylin-eosin and safranin-O. Each joint was scored for synovial hyperplasia (0–2), granulation tissue (0–2), fibrosis (0–2), degeneration of joint cartilage (0–0.5), osteoclasia (0–2), and osteogenesis (0–1). Histology score for each animal was the total of all the joint scores.

### X-ray score of bone erosion

The total number of joints in the proximal, middle and distal digital phalanges of the forelimb and hindlimb (second, third, fourth, and fifth digits) were assessed. When bone erosion was noted, 1 point was added to the score. The X-ray score for each animal was the total of the joint scores.

## 

HuizingaTWFleischmannRMJassonMRadinARvan AdelsbergJFioreSHuangXYancopoulosGDStahlNGenoveseMCSarilumab, a fully human monoclonal antibody against IL-6Rα in patients with rheumatoid arthritis and an inadequate response to methotrexate: efficacy and safety results from the randomised SARIL-RA-MOBILITY Part A trialAnn Rheum Dis201310.1136/annrheumdis-2013-20440524297381PMC4145418

## Notes

10.4161/mabs.28612
